# Supplementary material for: Underlying Role of Rumination-Mediated Attachment Style Plays in PTSD after TIA and Stroke
Source: Brain Sci. 2022 Aug 23;12(9):1118. doi: 10.3390/brainsci12091118 (PMC9497051; doi:10.3390/brainsci12091118)
Supplement: Supplementary file 1 [file brainsci-12-01118-s001.zip › brainsci-1801780-supplementary.pdf]

Supplementary Table S1 The demographic characteristics of 274 patients.

variables	number of people	No PTSD	Minor PTSD	Significant PTSD
Gender				
Male	186（67.9%）	59	112	15
Female	88（32.1%）	22	60	6
Degree of education				
Primary or below	104（38.0%）	20	76	8
Junior high	81（30.0%）	28	46	7
High school/technical secondary school	54（19.7%）	18	32	4
College degree or above	35（12.3%）	15	18	2
Professional status				
Farmer	120（43.8%）	37	74	9
Staff	73（26.7%）	33	37	3
Retiree	16（5.8%）	5	9	2
No work	65（23.7%）	6	52	7
Admission conscious state (awake)	265（96.7%）			
Ischemic stroke	253（92.3%）			
NIHSS scores (0-1)	125	46	73	6
(1-4)	93	28	57	8
(5-13)	35	3	26	6
Offending vessel				
anterior circulation	230	45	165	20
posterior circulation	23	22	1	0
Hemorrhagic Stroke	9（3.3%）	3	5	1
TIA	12（4.4%）	11	1	0

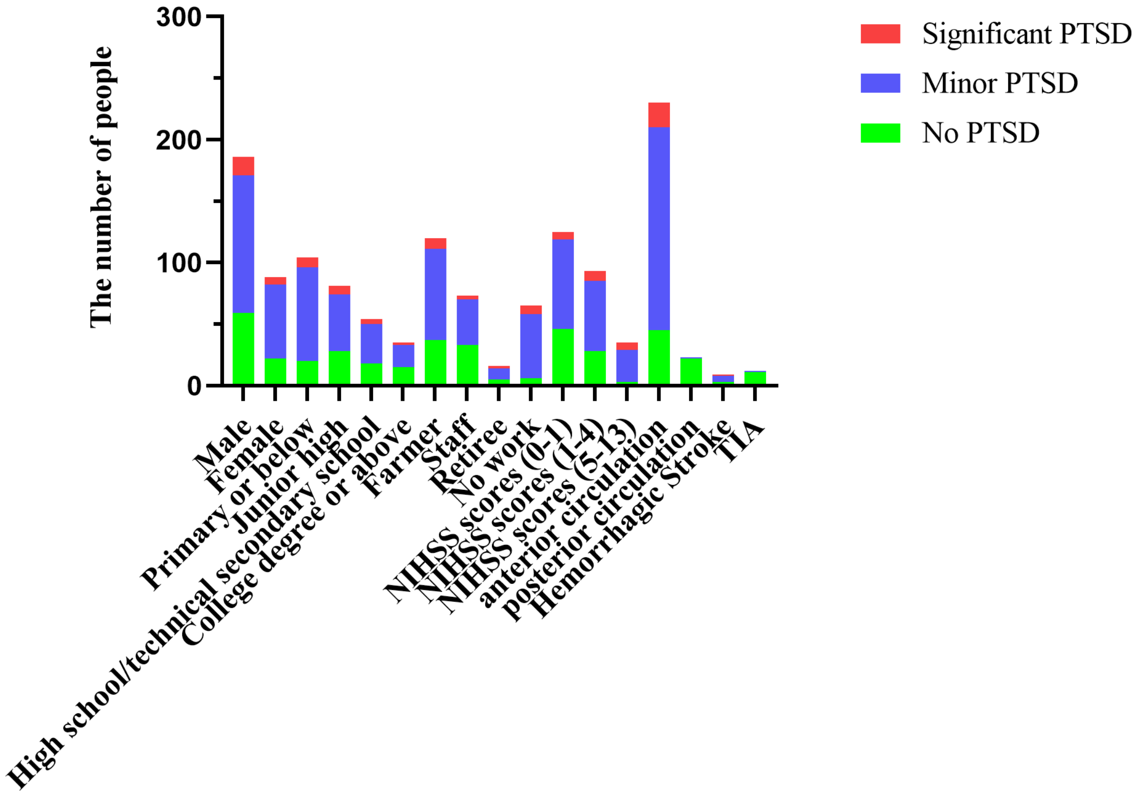

Supplementary Figure S1 The demographic characteristics of 274 patients.
